# Becoming Resilient: Promoting the Mental Health and Well-Being of Immigrant Women in a Canadian Context

**DOI:** 10.1155/2012/576586

**Published:** 2012-06-18

**Authors:** Judith A. MacDonnell, Mahdieh Dastjerdi, Nimo Bokore, Nazilla Khanlou

**Affiliations:** ^1^School of Nursing, York University, HNES Building, 4700 Keele Street, Toronto, ON, Canada M3J 1P3; ^2^School of Social Work, York University, Ross Building, 4700 Keele Street, Toronto, ON, Canada M3J 1P3; ^3^Echo Chair, Women's Mental Health Research, Faculty of Health, York University, HNES 3rd Floor, 4700 Keele Street, Toronto, ON, Canada M3J 1P3

## Abstract

This paper reports on grounded theory findings that are relevant to promoting the mental health and well-being of immigrant women in Canada. The findings illustrate how relationships among settlement factors and dynamics of empowerment had implications for “becoming resilient” as immigrant women and how various health promotion approaches enhanced their well-being. Dimensions of empowerment were embedded in the content and process of the feminist health promotion approach used in this study. Four focus groups were completed in Toronto, Ontario, Canada with 35 racialized immigrant women who represented diverse countries of origin: 25 were from Africa; others were equally represented from South Asia (5), Asia (5), and Central or South America and the Caribbean (5). Participants represented diverse languages, family dynamics, and educational backgrounds. One focus group was conducted in Somali; three were conducted in English. Constructivist grounded theory, theoretical sampling, and a critical feminist approach were chosen to be congruent with health promotion research that fostered women's empowerment. Findings foreground women's agency in the study process, the ways that immigrant women name and frame issues relevant to their lives, and the interplay among individual, family, community, and structural dynamics shaping their well-being. Implications for mental health promotion are discussed.

## 1. Introduction

The notion of empowerment as a feature of mental health promotion in Canada is not new. Over one decade ago, the Centre for Health Promotion at the University of Toronto, Ontario, Canada, [1] called for a positive view of mental health promotion aligned with the Ottawa Charter for Health Promotion's [2] priorities of community development, empowerment, and community capacity to promote equity and social justice. Immigrant women are one such population that experience health inequities. Although they are not a homogenous group, having different contextual supports and challenges, for the most part, their lives are represented in mainstream discourse as a homogenous population “in need,” in terms of their risks for disease, lack of access to culturally relevant services, and thus their burden on the health system. Yet, their vulnerabilities must be contextualized in relation to their status as racialized women who encounter other layers of resistance to support that include discrimination and/or marginalization of their health concerns [3]. Thus, accounting for empowerment in the generation of knowledge and practices relevant to their lives is an ethical imperative for researchers who advocate for them.

Nursing and interdisciplinary health researchers working within critical/feminist health promotion frameworks advocate creating opportunities for immigrant women as a population and set of subpopulations to create meaningful knowledge and responsive strategies to foster their well-being [4–7]. Certainly, given the recent political climate in Canada in which public sector policies and cuts in support for women-focused agencies and settlement programs and resources have significant impacts on women [8, 9], this focus seems even more urgent.

Minority populations bear a disproportionate burden of chronic disease including mental health concerns that have been attributed to complex social determinants of health along with the particular stresses related to minority status [10, 11]. Although gender is a critical determinant of mental health so that diverse women experience and understand mental health and the systems that address it in different ways, a focus on prevention is limited [12]. Canadian research on immigrant and racialized women's mental health points to culturally relevant support that reflects broad and holistic conceptualizations of mental health that move beyond Western-based medicine with its reductionist focus on diagnosis and treatment of psychiatric disorders (e.g., [7, 13–15]). A consideration of diversity of age and education, for example, within these groups of women is needed to promote effective prevention strategies.

Social determinants of health such as social exclusion, racism, employment, and poverty have been linked to immigrant women's well-being [6, 16]. An increasing body of research reflects the mental health implications of settlement that include ill health related to acculturation stresses including the family upheaval, social isolation, violence, addictions, language and cultural barriers, employment, certification processes, and lack of access to programs and services that many encounter (e.g., [6, 12, 17–20]), and gender is implicated in these migration processes [5, 6, 19–22]. Dynamics of stigma and silence in relation to mental health continue to pervade many Canadian communities including these diverse cultural immigrant groups. Despite an increasingly diverse population and large proportion of immigrants to Canada, our understanding of how to enhance the mental health and wellness of diverse immigrant women remains limited; nor has there been much attention to their contributions to the system in relation to promoting mental well-being.

The purpose of this paper is to report on grounded theory findings that are relevant to promoting the mental health and well-being of immigrant women in Canada. The findings illustrate the relationships among settlement factors and dynamics of empowerment that have implications for “becoming resilient” as immigrant women, as well as the relevance of various health promotion approaches to enhancing their well-being. Dimensions of empowerment were embedded in the content and process of the feminist health promotion approach used in this study.

## 2. Materials and Methods

This paper is based on focus group data collected in the Greater Toronto Area (GTA), a large city in Ontario, Canada, part of a larger qualitative policy study that aimed to understand activism as a feature of mental health promotion for immigrant women. We focus here on grounded theory findings that contribute to an understanding of “becoming resilient” in this context.

### 2.1. Feminist Health Promotion

 Consistent with feminist health promotion [23], which has goals of building community capacity, this study aimed to improve the everyday lives of diverse groups of immigrant women. We chose focus groups to spark interaction among participants in order to more deeply examine settlement experiences as the women themselves compared their stories with others in the group so that they could identify factors that shaped their lives and their actions in response to the settlement experience, that is, activism [24, 25]. A participatory and community-based research approach, a partnership between academics and a community agency, was chosen with goals of addressing social inequities and enhancing immigrant women's health using a strength-based focus to build on community knowledges and practices [26]. Study priorities included a commitment to enhancing the voices and visibility of immigrant women themselves and building in processes that foreground their agency and empowerment, all of which are key components of a feminist health promotion approach. In a similar vein, attention to gender and its intersections with race and other social differences required examination of the complex dynamics of power and privilege that shape community-academic collaborations across all dimensions of the study, from conceptualization to data collection and analysis and knowledge mobilization. The focus on mental health promotion and policy change was consistent with our partner community agency's priorities and also with the feminist health promotion priority on social action as an outcome of the research [23].

The project was conceptualized, and all phases of the research occurred in partnership between the researchers at York University School of Nursing in Toronto and community partnering agency Women's Health in Women's Hands Community Health Centre in Toronto, whose mission is to serve women from Africa, South America, and Caribbean and Asian communities including immigrant women. All except one member of the research team, who was a second-generation European immigrant, were immigrants, having arrived in Canada at least ten years ago.

### 2.2. Constructivist Grounded Theory: Sampling Strategy

This qualitative study was based on constructivist grounded theory methodology. This approach offers a substantive theory grounded in the findings that can foster understanding of the processes embedded in a phenomenon of interest, in this case, the relationships among settlement, activism, and immigrant women's well-being [24]. Theoretical sampling using a constant comparative approach directed participant selection and was used to elaborate and refine categories informing the theory which emerged. Given these inductive processes, sampling, data collection, and analysis were concurrent. Constant comparison of incidents and categories was carried out, and when no further information was added through analysis, saturation occurred; thus, sampling was complete [24].

### 2.3. Study Procedures

Ethics approval was received from York University Office of Research Ethics. To participate in the study, women were to be over the age of 18, living or working in the GTA, and either immigrants residing in Canada for a minimum of three years or those who had completed the refugee process. Recruitment of participants occurred through the agency's extensive networks; online copies of study flyers were shared through listserves, and hard copies of study flyers were posted in the agency and in agencies serving immigrants in Toronto. Participants were directed to contact the study coordinator to participate. Arrangements were made to find a time and location for focus groups that would suit participants. Aware that these women often juggle work or family demands and may have limited time to participate and may be living in poverty, a small cash honorarium was provided to all participants.

Shortly after the study was posted, a key informant from the Somali community expressed interest in the study and met with two researchers on the team in order to discuss the nature of the study. She shared information on the study with women through her networks. The first focus group was completed by a researcher who facilitated discussion in the Somali language. For this focus group, all information postings and consent forms were translated into Somali, explanations related to the study for the informed consent process were in Somali, and the audiotaped transcript was translated into English for analysis. For reasons of feasibility, the other three focus groups were conducted in English. Participants' facility with spoken and written language varied considerably. In all focus groups, study information was provided in written format and researchers reviewed study information and points on the informed consent form verbally with each participant as part of the informed consent process. Researchers stressed the voluntary nature of participation and the researchers' plans to protect identifying information. Participants were asked not to share information shared within focus groups, but researchers cautioned that confidentiality could not be guaranteed. Given the sensitive nature of the information that could be shared in the focus group, participants were given information about counselling and other resources that were available. Participants received a copy of the signed consent. After signing the informed consent form, participants individually shared brief demographic information verbally or on a written form. Two or three researchers facilitated and digitally recorded 90-minute focus groups. There was a primary facilitator, and the other researcher(s) managed instrumental tasks such as the tape recording, obtaining consent forms, and timekeeping. They also participated in the dialogue and interview questions or probes as per the feminist health promotion principles that encouraged participatory team involvement. We followed Charmaz's [24] view of the active participation of the researcher in the focus groups in co-constructing the way the stories were emerging. As she says, the researcher remains active in interviewing, “alert to interesting leads” (p. 32). Focus group questions followed a semistructured interview guide and probed women's everyday lives, settlement, immigration experiences, and health/community connections. No identifying information was shared in reporting here. Narrative excerpts are linked to the respective focus groups (FGs).

### 2.4. Participant Sample

These four focus groups with 35 immigrant women living in the Greater Toronto Area (GTA) occurred from December, 2009 to July, 2010. The majority of participants self-identified as visible minorities (22/35); several also self-identified as women living with disabilities and/or sexual minorities. They represented diverse countries of origin: 20/35 were from Africa, and the others were equally represented from South Asia (5), Asia (5), and Central or South America and the Caribbean (5). Their first language and ethnocultural backgrounds included South Asian, West Indian, Asian, African, and South American women including those from Uruguay, Nepal, China, Jamaica, Ethiopia, Somalia, Uganda, India, and Pakistan. Participants' education and facility with spoken and written English varied considerably, even if they spoke several languages; all but two identified a language other than English as their first language. These participants represented women ranging between 25 and 65 years of age; most were between 45 and 54 years of age.

### 2.5. Grounded Theory: Concurrent Sampling, Data Collection, and Analysis

We began sampling with a focus on immigrant women's settlement experiences and how they responded to everyday challenges. As Charmaz [24] states, “Initial sampling is where you start … establish sampling criteria for people, cases, situations” (p. 100). Initially we recruited Somali-speaking women for FG no. 1. As per the constructivist grounded theory approach, transcripts were coded line-by line and/or incident to incident. As an example of the latter, an incident of everyday adjustment to the weather for one woman could be contrasted with an incident of adjustment to the new neighbourhood for another. From this FG no. 1, a category of settlement experience emerged which raised questions about multiple factors that were affecting their settlement experience such as their age, family dynamics, educational and cultural background, and employment opportunities.

We then used theoretical sampling, which according to Hood as cited in Charmaz [24] indicates that “theoretical sampling is … purposeful sampling according to categories that one develops from one's analysis and these categories are not based upon quotas; they're based upon theoretical concerns” (p. 101). Thus, for theoretical sampling of participants in the focus groups that followed the first focus group,we aimed to gather women who were diverse according to these identified settlement factors such as language, country of origin, and ethnocultural background to hear their settlement stories and better understand whether and how these factors influenced their settlement experiences and capacity to take action. Constant comparison of incidents and categories was carried out, and when no further information was added through analysis, saturation occurred; thus, sampling was complete at the FG no. 4 [24].

What emerged from constant comparison of these categories and incidents of settlement was not just these factors, but also new categories which reflected women's empowerment through taking action. These new categories pointed to a set of phases linking women's experiences and actions, and a core category emerged as relevant during FG no. 3. We continued to apply constant comparison. At the end of these four focus groups, analysis indicated that saturation had occurred, and no further information was added for this core category [21].

Over the course of this analysis, a critical lens was applied that takes into account the historical, economic, political, and sociocultural dynamics that shape people's lives, such that everyday dynamics of power and privilege related to dynamics of gender and race and other social difference, were integral to the analysis [4]. As the analysis shows, constant comparison of categories and subcategories created a substantive theory about “becoming resilient” that was grounded in the findings [24].

### 2.6. Rigour

To ensure rigour, the transcripts were analyzed independently by at least two members of the research team who noted emerging categories through a process of memoing [24]. The researchers listened to the digital recordings on several occasions and as a team reflected on the emerging categories and the implications for co-creating the realities of these women's lives in ways that would honour and represent their voices and visibility. Thick description can enhance transferability of the findings. Thus, narrative excerpts are also used to report findings. This reflexive process offered many opportunities for the research team to reflect on how they were situated with respect to the topic and participants [23]. Identifying information was removed after transcription to protect privacy and confidentiality during the analysis phase and in reporting of findings.

It was not feasible to completed member checking in a way that focus group participants would review transcripts because of the nature of the focus group discussion and variability of English language literacy. However, upon completion of the full study, a community forum which was open to community members, health care providers, and other stakeholders was held at the partnering community agency, whereby the team shared findings and invited open discussion. The forum was attended by a range of community members including a number of focus group participants. Many attendees spoke of how the findings reported here resonated with their own experiences. Such strategies of prolonged engagement with participants, creating opportunities for ongoing researcher reflection, as well as documentation of an audit trail, which would enhance rigour through confirmability, dependability, and transferability, were also congruent with ethical principles of feminist health promotion [4, 24].

As noted, this was a reflexive process and team members reflected on their own settlement experiences and social location and how this influenced the analysis. The fact that the team members represented women, both insiders and outsiders to experiences of settlement and activism, was an asset for opening space for dialogue among participants. The team collectively represented a rich understanding of the complex dimensions that could be associated with settlement, activism, and well-being in terms of co-constructing the focus group data collected and analyzed [24].

### 2.7. Limitations

This qualitative study was not intended to be generalizable to the diversity of immigrant women in Canada, but to enhance understanding of their everyday lives. Given the focus on creating a purposeful sample, these findings reflect the voices of women who were interested and able to participate and the feasibility of providing opportunities for translation [27].

## 3. Results and Discussion

There was a great interest in this study by a variety of immigrant women as reflected by waiting lists for focus groups; participants were eager to share their stories. Analysis of findings indicates that multiple and complex settlement factors shape these women's mental health and well-being. In summary, the analysis pointed to the processes that shape resilience as a feature of mental well-being for immigrant women. These included facing everyday challenges (negative and positive experiences such as feeling hopeless and/or bouncing back from this negative feeling, i.e., resilience); naming these issues (e.g., language barriers, discrimination); framing them in terms of whether these reflected a biomedical, behavioural, or socioenvironmental health promotion perspective in order to take action. The set of categories that reflected the impacts on the women themselves of taking action (being involved in activism) at the individual or community level, for example, coalesced in a core category of “becoming resilient.”

 In the following section, we first detail factors implicated in the settlement processes that contribute to immigrant women's experiences and actions, linking these to resilience with attention to two categories: *immigrant women facing everyday challenges in the settlement process*; and *naming and framing the issues*. Implications for immigrant women in terms of creating conditions that foster empowerment and understanding of how they express their agency, taking action and broader implications for mental health promotion, are then discussed.

### 3.1. Immigrant Women Facing Everyday Challenges in the Settlement Process

Although each group reflected a unique dynamic of women across difference with respect to language, age, and cultural affiliations, the focus group of Somali women offered a particularly sensitive dynamic. It was comprised of women from the war-torn Horn of Africa (e.g., Somalia) whose lives and identities are inscribed by tribal affiliations, traumas, and violence from years of civil war. While we had planned for 8 participants, 15 arrived, representing diversity in modern through traditional ethnic dress and eager to take part in this forum. This was despite tribal differences and traumas that many women in these communities have faced and that have created barriers for them to come together. All participants were stirred by this opportunity to share in the tasty community-sponsored potluck meal that preceded the formal focus group and listen with openness as two participants read the informed consent to the others in Somali and then opened the discussion sharing their stories, ending with a unity song. On this day, this particular group of women put aside their home country's politics and tribal divisions and started to discuss openly their thoughts, feelings, hopes, and dreams without fear of judgment. Afterwards, one participant remarked.

This group of women comes together from time to time to support each other, and discusses common issue such as settlement difficulties, sharing information/ideas and helping those in need, but they never openly discussed individual pains and sorrows as they did today. (FG no. 1)

Such dynamics which emerged from this first of our focus groups set the tone for forums that had the potential for empowering impacts beyond the transcribed words. Certainly the three focus groups that followed were more heterogenous with respect to ethnocultural representation.

However, in each of the focus groups, participants were keen to share details of their of everyday settlement experiences. Women spoke about challenges and barriers to accessing services, care, housing, education, employment, language, and credentialing, plus family concerns often related to changed gender roles in addition to many other dynamics. Through this process of narrating stories and having opportunities to describe their own lives and learn about issues common to others, the focus groups fostered spaces to affirm the emotional work of settlement that can be, in itself, empowering and therapeutic. As a number of women indicated explicitly, they found the focus group a safe environment. Certainly such moments reflect a spiritual dimension to the study processes that reflects support for women to make meaning of their lives, and that is also consistent with a sense of connectedness that spirituality can represent.

Narratives reflected many factors which affected participants' capacity for well-being. To enhance voices of diverse women, show the complexity of their everyday experiences, and offer a flavour of these everyday challenges, examples of verbatim narratives are used in the next sections. Challenges encompassed concrete factors such as lack of knowledge, disabling health conditions, social isolation, family roles, age, language, or financial barriers as well as social dynamics such as identities and discrimination.

Social support and isolation were embedded in many narratives. We focus here on narratives that link their well-being to social isolation and provider, family, and community support. In this first example, a woman who struggles with depression talks about having no relationships with neighbours or family such that she is unable to locate basic information about health services when she becomes ill and struggles to find health system support for her illness.

I was very sick … too weak to go to the hospital … not eating … back home the doctor comes home. I was then 3 days in the emergency room. They did not take me to a room … it was terrible listening to everything …. So my experience with health system not very good. Even I call places and say, “Please, I am alone.” (FG no. 4)

Isolated parents, whether they were partnered or raising children on their own, at times struggled with language barriers that exacerbated their capacity find effective support from providers. A mother who had become pregnant with her second child shared.

I said, “This is so crazy …. I have to do something, like not just sit down and raise a child. So I do not want to feel guilty that I put them in daycare.” … and I feel depressed … like losing my mind. Like the baby is crying all the time and I feel like throwing her. Somebody take *[sic]* her. Like I feel inside there is no family to help you and talk to. And nurse used to come and she does not understand me and she's trying to encourage me saying “Breastfeed her.” I say, “I will.” and “Just go.” (FG no. 4)

Participants spoke generally about the impacts of stress on their lives and families. As a number of them indicated, however, they are hopeful and use a variety of strategies to stay healthy which include tapping into their inner resources and contributing to the development of community and neighbourhood support. As one woman remarked.

I always exercise and I do the meditation everyday … the second thing is very important is to help people … our neighbour, our family, our city, our country … help always brings satisfactions. And study, continuous study …. I think even though the situation has not been easy we have to be in good attitude always to be positive. (FG no. 4)

Another spoke of relaxing by “just listen[ing] to my radio stations back home. Just do stuff to say I am not there physically, but spiritually I can still find out things are going on …. Just try a coping mechanism that's what I did” (FG no. 4). The links between stress and physical well-being were noted as a young mother shared.

As for me, I was in swimming before. After coming here, having babies, I have no time to exercise. My husband buy me treadmill and still do not have time to use it for two years …. I gained lots of weight. I weighed 200 lbs because of stress. After taking classes I feel happy and my situation improved and lost weight. (FG no. 2)

A number of them talked about their individual experience of trauma and violence in war-torn countries prior to settlement, as well as after settlement, related to family violence and discrimination based on health status such as being HIV positive. As each woman discussed the challenges for her community, she highlighted how individual barriers are affected by the community and structural supports more broadly, as described in the following section.

### 3.2. Naming and Framing the Issues

It was clear that as they described everyday challenges as individual immigrant women, as members of families, diverse communities, and larger societies, participants across the lifespan situated these individual factors in the larger family, community, and social contexts. The participants' narratives moved beyond description to analysis.

 Family networks were identified as one of the most accessible settlement resources. Participants described family in a variety of configurations as close and distant kinship networks including but not limited to tribal relations from the country of origin, female-headed households that encompassed both heterosexual and same-sex relationships. Despite the stressful nature of immigration, resettlement dynamics were often mediated in positive ways in relation to the availability of a supportive family. Women's perceptions of instrumental support such as food, shelter, and emotional support varied depending on the woman's immigration status, class background, sexuality, family configuration, and reason for immigrating to Canada.

Effective support for one new mother was reflected in her male partner's offer to seek a survival job while she looked after their infant. Intergenerational support was noted by an older Somali woman who found emotional and financial support from her grandson so that she could find an apartment away from social housing. “Thank God, he turned out to be a good boy. He completed high school … is saving money to attend university. He helps me financially as well. Alhamdulilah” (FG no. 1). Some women reunited with their husbands in Canada. A number with a supportive partner found this journey enjoyable in spite of the stressful nature of immigration, while others experienced isolation and unexpected upheaval that can emerge with the changed gender roles upon migration to Canada. As a young Asian woman shared.

My husband also faces health problem because he have *[sic]* to go to work and has tension. I am very sensitive, start to cry when I talk to him and he give me confidence. He shows me he is strong, but he is not …. So men and women both affected. (FG no. 2)

Those with inconsistent or no supportive family, especially without other community connections, felt emotional stress. A number of them shared stories of grief, family disruption and violence which resulted in encounters with the health and social service system. In the following excerpt, a mother describes the overwhelming grief that underpins her everyday capacity to face life and work, identifying patience and silence as strategies for survival in the face of ongoing frustration with career opportunities.

It is not easy. It is almost six years …. My son he got in an accident. I was work[ing] when they said to me that your son is dying …. He died under the car …. Instead of supporting me they blame me, painful, painful wherever you go …. So I have to do whatever I can do. Patient is more than anything that is important, otherwise … stress will kill you …. Almost you blow out your brain. You cannot save yourself. You cannot save your children. You cannot *[sic]* do nothing, paralyze you …. Do what you have …. I have Masters degree, but I … work at hospital, cleaning. I hide my career, to survive …. I recommend patience to everyone. (FG no. 3)

Immigrant women's culture, precarious immigration status, language barrier, and lack of networking in the postmigration setting increased their vulnerability for being abused. Women living in poverty who dealt with violence from abusive husbands who were also dealing with addictions and past experiences of war-related trauma were at risk for mental health problems as illustrated by the following participant. She arrived in Canada with her two sons to be reunited with her husband, only to encounter violence not just from him, but also within a few years from one son in the cycle of violence that plagues the family within these traumatized communities. She shared.

He wanted to assist us, but he was having drinking problem …. When I found out …. I decided to leave him …. On one occasion he threatened me with violence and said, “I will cut you into pieces.” I experienced fear, trauma …. He also tried to turn the boys against me … accused me for neglecting our boys—leaving them all alone in the house. He made complaints against me and took such complaint to Children's Aid which found such allegation baseless …. This really affected me …. I suffered insomnia for 10 years. There were times that I felt that I was going to lose my mind. (FG no. 1)

Another dimension of family support was illustrated by an older South Asian woman who talked about her living conditions. She had been sponsored by her daughter and had the role of caring for her active preschool and school-aged grandchildren and cleaning the house. “Always I am in mental depression. My husband and me always …. I live with my daughter. She has three bedrooms, but …. I live in one bedroom … my husband and me. Very difficult for me” (FG no. 4). Whether the daughter was aware or not of the impact of living conditions on her mother, both older and younger members of the family were affected by social policies that limit childcare support in ways that older immigrant women often assume child-minding responsibilities.

Lack of childcare was frequently noted as a crucial barrier for completing required education and credentialing programs, as well as for job searches and employment. One participant needed childcare to continue a part-time job but was refused.

The case worker … said, “No. Either you get a full time job by [this date] or your children will not get child care. I cried for a few days, and thought, “I cannot find a full time job. I am almost in the position to do good things for community and how the system is killing us.” I cannot say anymore than this. I have no word. (FG no. 3)

Women frequently made links between employment and underemployment in ways that point out the ongoing diminishment of immigrant women's knowledge and skills and the racialization processes that structure their lives in ways that focus on language, job training, and volunteer opportunities with implications for families' physical and mental well-being. As a participant remarked.

And always in my country I am more intelligent. I am member of …. International Lions Club. And I help many people and I volunteer in camp, free eye camp. Full day I am working and volunteering here and there. I enjoy very much my country … but few time I am in depression …. But now joined many program *[sic]* in my neighbourhood here and there then I am a little bit okay now. (FG no. 4)

Underemployment surfaced frequently as a stressor that interacted with financial hardship, credentialing, and gender to shape their lives. In the following excerpt, a single mother identifies mental health concerns while she raised a family and engaged with higher education. She links her individual well-being to dynamics of family roles and expectations that shift with settlement in the new country. Her well-being cannot be divorced from the ways in which her life is inscribed by traditional cultural expectations that place the responsibilities of informal caregiving and unpaid work for children and elders on women. Nor can it be understood, as she points out, without taking into account the lived realities of inadequate Canadian health and social policies that have similar impacts and which exacerbate stresses for all family members rather than supporting their well-being and rights to live with dignity. The intersections between age, gender, poverty, employment, and immigration status are clear.

I had to deal with depression/anxiety, being the head of my own house, two kids and so much debt because my parents could not afford it …. My mom, professor, and dad, a dentist, and they come here and my dad is cleaning toilets and my mom is struggling getting community work. So just trying to balance with money and housing, paying medication that is not covered. That whole experience is so disgusting. You learn all the benefits that Canada has and you are living the experience where it does not apply to you, where I make a bit too much money … will they cut back on my health [benefits]? (FG no. 4)

One participant makes links between her health and finances, housing, and underemployment and notes it has implications for her role in caring for others in the family.

You have to do two or three jobs to maintain family … pay rent which is high. You cannot afford to buy proper food, to eat proper food, the time to eat. Then you end up having high blood pressure, diabetes, high stress …. You end up eating food with sugar and carbohydrates and end up obese. Right? And with obesity you have high blood pressure, heart disease, diabetes and other things …. So it all comes back to the stress … to under employed and health problems. (FG no. 2)

Another participant foregrounds the endless maze of programs that await them as they adjust to the migration experience, having concluded that the answer requires attention at the structural and policy levels.

All settlement workers have problem with clients who have stress and unemployment. If the government solved employment and education … the mental health problem will be solved …. Focus group is not needed …. Very frustration *[sic]* for me. I have master degree, ten years experience. I did immigrant women integration program and every time I finish one course I do another one …. They say I need confidence. Not that I am shy or lack confidence …. I go to interview. For some reason they do not want to accept high qualified people …. What can I do? The main issue of mental health for immigrant is employment program. (FG no. 2)

These women's narratives thus address structural barriers to access to housing, education, employment, and information about available services as determinants of their own mental well-being and also with implications for their families and cultural communities and neighbourhoods. In the following example, a participant attributes the recent onset of mental and physical ill health in part to poor housing conditions that focuses on her individual experience, but shows how she, as others have in earlier examples, takes action to improve her situation, showing how they are resourceful and become resilient in the face of adversity.

My immune system start going down especially in the last two years …. I got depression and lots of physical problems I never had in my life and I … look on internet and trying to get better you know? …. I do not like to take a lot of medications so I am with a naturopathic so I feel much better. (FG no. 4)

In contrast, women who came from war zones talked about the similarity of marginalized neighbourhoods in Toronto to the war zones they came from. Having knowledge of someone who has lost a child to guns in marginalized neighbourhoods or losing their children to the justice system makes them feel unsafe and violated all over again. They discussed community members who are struggling with access to safe housing, appropriate services, and dealing with the financial burden that will affect their communities for years to come.

In this paper, we foreground empowerment in terms of the content and process of a study whose findings illustrate how immigrant women in this large Canadian urban metropolis experienced and understood their settlement and its relationship to their well-being. The chosen grounded theory methodology and lens of analysis show how this strengths-based approach offers insight into empowering dynamics that foster understanding of mental well-being as women themselves articulate this, processes that can contribute to resilience that is often identified in strengths-based studies with immigrant women [7, 12]. Such dynamics are congruent with a notion of resilience as described by Joubert and Raeburn [28], the capacity to “cope with, and bounce back after, the ongoing demands and challenges of life, and to learn from them in a positive way.”

In contrast to dominant deficit-based discourses about immigrant women, these findings illustrate how immigrant women are knowledgeable and articulate and critically conscious who implicitly and explicitly point out the links between their individual experiences and larger family, community, and social structures. Consistent with Guruge and Khanlou [29], even when participants are identifying individual-level practices, they are often referring to dynamics that require attention at the family, community, or structural level, as these authors use an ecosystemic framework. Processes which promote mental health can also promote resilience and vice versa; these require attention to multiple dynamics of well-being that include individuals, families, communities, and societies and the ways they interact [30].

It is evident that these participants shared important aspects of their settlement processes that resonate with much of the literature on the challenges faced by immigrant and many refugee women. These findings support and extend studies with diverse Canadian immigrant women that identify settlement dynamics as contributors to mental health through acculturation processes, family upheaval, social support/isolation related to family, community, or other networks, and discriminatory processes (e.g., [7, 13, 17, 31–34]). In contrast to studies which focus specifically on one cultural group, this study brings together a diverse ethnoracial sample with a goal of understanding common processes and experiences.

The chosen methodology, with its principles of feminist health promotion, has potentially empowering impacts for the women themselves. The study foregrounds the strengths that the women themselves bring, as well as the processes that can enhance their well-being. Their enthusiasm for participating in collective story telling and sharing reflects earlier research with marginalized women, such as immigrants, for whom focus groups can be a vehicle for social support, fostering community ties for isolated women [4]. Describing individual stories in a collective space can validate women's perceptions and understandings of their lives and knowledge that are often discounted or rendered invisible in relation to dominant views, especially for racialized women [35].

These narratives reflect a variety of understandings of health and mental health that reflect philosophies that are informed by biomedical, behavioural, and socioenvironmental lenses, each of which demands a set of different strategies for health promotion and thus mental health promotion [36]. For the most part, an explicit focus on mental illness was limited in these narratives. With few exceptions, participants used the term “stress.” For some, mention of depression or anxiety was also linked to their search for relevant health services that would provide treatment for mental illness. Although this biomedical lens with its focus on diagnosis and treatment is important, effective support for mental well-being requires a broader understanding that reflects a more holistic view of mental health [7, 14, 15]. Similarly, a view of mental health which aims to have individuals make changes in behaviour or attitudes, such as a focus on coping skills or reframing issues, puts the responsibility for well-being at the individual level. Consistent with Ahmed et al. [13], a number of participants did find preventive practices at the individual level helpful, identifying the need to have positive attitudes, eat healthy food, exercise, and use effective coping skills, actions consistent with behavioural approaches to health promotion [36].

However, if strategies for change, that is, promoting mental health, are exclusively focused on biomedical and behavioural understandings of well-being, there is not only the possibility of blaming the victim, but also limiting effective solutions that address structural change. Certainly the issues identified by the Somali focus group suggest that little has changed for the better over the last decade [32, 37]. A socioenvironmental lens, which would include attention to both the processes and content of strategies such that communities themselves identify solutions and take leadership in creating strategies, offers the possibility of mental health promotion that can address inequities as well as foster conditions and processes that enhance the spiritual dimensions of immigrant women's lives [14, 38]. In these narratives, participants often situate individual immigrant women's well-being in the larger family, community, and structural contexts, naming social determinants of health as key to their lives and what Guruge and Khanlou [29] consider “intersectionalities of influence.” Complex dynamics at the micro-, meso- and macrolevels shape diverse immigrant women's mental well-being in particular ways in relation to social, political, economic, historical, and cultural contexts [39].

## 4. Conclusion

This study offers insight into the mental health promotion context of settlement issues for immigrant women in Canada whose voices are often unheard, including Somali women and a range of ethnically diverse women, for whom many languages other than English are their first language. It is clear through their narratives, however, that despite the multiple and often overwhelmingly deeply entrenched barriers to achieving wellness that they encounter in their everyday lives, they show resilience and strength as they interface with others at the individual, family, community, and system levels.

The findings support the creation of health promotion initiatives that are consistent with a population health promotion approach that is congruent with a socioenvironmental approach and takes into account empowerment processes and social determinants of health [4, 40]. Certainly, the current Canadian Community Health Nursing Standards of Practice with their focus on equity through relational practice and building of community capacity support nursing practice to promote populations and communities health [41, 42]. Given the predominance of medical and behavioural models that inform clinical practice in relation to mental health and illness, as well as health promotion practice for nurses and other health professionals, these findings support calls for attention to comprehensive approaches and upstream measures including policy that shape conditions which enhance immigrant women's and other communities' resilience [7, 12, 39, 42].

A focus on creating evidence for mental health promotion is timely, given the multiple factors and values that must be taken are relevant to the enterprise of evidence for policy making [43]. Findings from this study suggest that such mental health promotion strategies would (1) build on knowledges processes, and resources that immigrant women have created, (2) take account of the ways that knowledge about the mental health promotion of diverse populations is created, and (3) encompass comprehensive strategies such as creating supportive environments and enhancing participatory processes for policy changes that are needed to foster the mental health and well-being of diverse groups of immigrant women. There are implications for nursing and interdisciplinary researchers and practitioners to consider how they are implicated in promoting the mental health of the communities they serve.
